# Effects of Silica and Titanium Oxide Particles on a Human Neural Stem Cell Line: Morphology, Mitochondrial Activity, and Gene Expression of Differentiation Markers

**DOI:** 10.3390/ijms150711742

**Published:** 2014-07-02

**Authors:** Kouki Fujioka, Sanshiro Hanada, Yuriko Inoue, Keisuke Sato, Kenji Hirakuri, Kouichi Shiraishi, Fumihide Kanaya, Keiichi Ikeda, Ritsuko Usui, Kenji Yamamoto, Seung U. Kim, Yoshinobu Manome

**Affiliations:** 1 Division of Molecular Cell Biology, Core Research Facilities for Basic Science, The Jikei University School of Medicine, Tokyo 105-8461, Japan; E-Mails: kfujioka@jikei.ac.jp (K.F.); ikedak@jikei.ac.jp (K.I.); ree-chicochamaro@jikei.ac.jp (R.U.); 2 Research Institute, National Center for Global Health and Medicine, Tokyo 162-8655, Japan; E-Mails: hanada@ri.ncgm.go.jp (S.H.); fkanaya@acc.ncgm.go.jp (F.K.); ykenji@hosp.ncgm.go.jp (K.Y.); 3 Department of Anatomy, Toho University, Tokyo 143-8540, Japan; E-Mail: yuriko.inoue@med.toho-u.ac.jp; 4 Department of Electrical and Electronic Engineering, Tokyo Denki University, Tokyo 120-8551, Japan; E-Mails: satok@mail.dendai.ac.jp (K.S.); hirakuri@mail.dendai.ac.jp (K.H.); 5 International Center for Materials Nanoarchitectonics, National Institute for Materials Science, Ibaraki 305-0044, Japan; 6 Medical Engineering Laboratory, Research Center for Medical Science, The Jikei University School of Medicine, Tokyo 105-8461, Japan; E-Mail: kshiraishi@jikei.ac.jp; 7 Medical Research Institute, Chung-Ang University College of Medicine, Seoul 440-746, Korea; E-Mail: sukim@mail.ubc.ca; 8 Division of Neurology, Department of Medicine, University of British Columbia, Vancouver, BC V6T 2B5, Canada

**Keywords:** silica, titanium oxide, nano, toxicity, neural stem cell, neural progenitor cell, differentiation

## Abstract

Several *in vivo* studies suggest that nanoparticles (smaller than 100 nm) have the ability to reach the brain tissue. Moreover, some nanoparticles can penetrate into the brains of murine fetuses through the placenta by intravenous administration to pregnant mice. However, it is not clear whether the penetrated nanoparticles affect neurogenesis or brain function. To evaluate its effects on neural stem cells, we assayed a human neural stem cell (hNSCs) line exposed *in vitro* to three types of silica particles (30 nm, 70 nm, and <44 μm) and two types of titanium oxide particles (80 nm and < 44 μm). Our results show that hNSCs aggregated and exhibited abnormal morphology when exposed to the particles at concentrations ≥ 0.1 mg/mL for 7 days. Moreover, all the particles affected the gene expression of *Nestin* (stem cell marker) and neurofilament heavy polypeptide (*NF-H*, neuron marker) at 0.1 mg/mL. In contrast, only 30-nm silica particles at 1.0 mg/mL significantly reduced mitochondrial activity. Notably, 30-nm silica particles exhibited acute membrane permeability at concentrations ≥62.5 μg/mL in 24 h. Although these concentrations are higher than the expected concentrations of nanoparticles in the brain from *in vivo* experiments in a short period, these thresholds may indicate the potential toxicity of accumulated particles for long-term usage or continuous exposure.

## 1. Introduction

Recent technical advances have enabled mass production of various nanomaterials, such as silica, titanium oxide, and carbon nanotubes. Although these nanomaterials are currently used in products that directly contact the human body, e.g., cosmetics [1,2] and food [3,4], their safe usage is still under investigation. In particular, there is a concern that some characteristics of nanomaterials, such as their tube- or fiber-like structures with rigid properties or certain sizes, might cause toxicity similar to that of asbestos [5,6,7].

Both *in vitro* and *in vivo* studies of nanoparticles toxicity are currently in progress [8,9,10,11,12,13]. *In vitro* studies have revealed several cytotoxic mechanisms, such as (1) reactive oxygen species (ROS) generation by cells that uptake titanium oxide particles [14,15] or silicon/silica particles [16,17]; and (2) the release of metallic material from Cd/Se quantum dots (QDs) after UV exposure [16] or silver particles [18]; and (3) structure-related toxicity caused by multi-walled carbon nanotubes [19]. Moreover, *in vivo* studies have revealed (1) alterations in blood components by the exposure of titanium oxide particles [20] or silver particles [21]; and (2) the distribution of QDs in several tissues [22,23,24].

Nanoparticles accumulation in brain tissue has also been described in many studies [22,23,24,25,26,27,28,29]. For example concerning QDs, intravenous injection of QDs coated with –OH, –NH_2_, or –COOH functional groups results in different rates of brain penetration [22]. Furthermore, in a pilot study, cadmium ion was slightly detected in the brain tissue of rhesus macaques after the injection of phospholipid micelle-encapsulated CdSe/CdS/ZnS QDs [24].

Other studies showed that the penetration of nanoparticles into the brain differs depending on their size [21,29,30]. The silver particles smaller than 100 nm (22, 42, and 71 nm) have been demonstrated to penetrate into the murine brain, whereas 323-nm particles have not been found in the murine brain [21]. Moreover, intravenous administration of 70-nm silica particles in pregnant mice resulted in placental penetration and accumulation in the fetal brain, whereas 300- and 1000-nm particles did not cross the placental-maternal barrier [29]. Our previous study also showed size-dependent penetration of silica particles with a blood-brain barrier model *in vitro* [30]. The apparent permeability coefficient (Papp) in the model for the 30 nm silica particles was higher than those of the larger silica particles (100 and 400 nm) [30]. These reports indicate that some nanoparticles, especially the particles smaller than 100 nm have the potential to penetrate brain tissue.

However, few *in vivo* experiments have revealed how nanoparticles affect brain functions. Because *in vivo* assessment of brain functions involves many aspects, such as neural activity, brain tissue inflammation, and behavioral evaluation, it is difficult to evaluate the functional effects of a small number of particles on the brain. 

Therefore, for evaluating the effects on neural development or brain function, we investigated the effects of nanoparticles on neural stem cells (NSCs). NSCs are precursor cells that develop into neurons and glial cells in the fetal brain during embryonic development [31]. Furthermore, recent reports indicated that NSCs also exist in the adult brain, specifically in the subventricular zone and the dentate gyrus of the hippocampus, and are responsible for neuronal regeneration [32,33]. Another study showed that high mobility group AT-hook (HMGA) proteins have been reported as a factor in fate transition or restriction of neural precursor cells [34]. Thus, the investigation of NSCs activity will be helpful in evaluating the effects of nanoparticles on neural development or brain function.

As for nanoparticles’ effects on the human NSCs (hNSCs), a few *in vitro* studies using cell lines have been reported [35,36]. Song *et al.* showed that proliferations and viabilities of hNSCs were not affected by the co-culture of some superparamagnetic iron oxide nanoparticles (around 28/100 nm) at 25 μg/mL for 24 h [35]. In another study, Söderstjerna *et al.* reported a significant effect on the sphere size- and morphology of human embryonic neural precursor cells was found for all cultures exposed to gold and silver nanoparticles (20/80 nm) at 50 or 800 particles/cells, although these particles did not significantly affect the total number of living and dead cells [36]. Both studies investigated the effects at lower concentration ranges and left possibilities of further investigations for potential toxicity at higher concentrations.

In this study, we revealed toxicological effects and their threshold concentration of nanoparticles on human NSCs (hNSCs) line using three types of silica particles (SP), SP30 (30 nm), SP70 (70 nm), and SPM (<44 μm), and two types of titanium particles (TP), TP80 (80 nm) and TPM (<44 μm).

## 2. Results

### 2.1. Physical Properties of Particles

**Figure 1:**
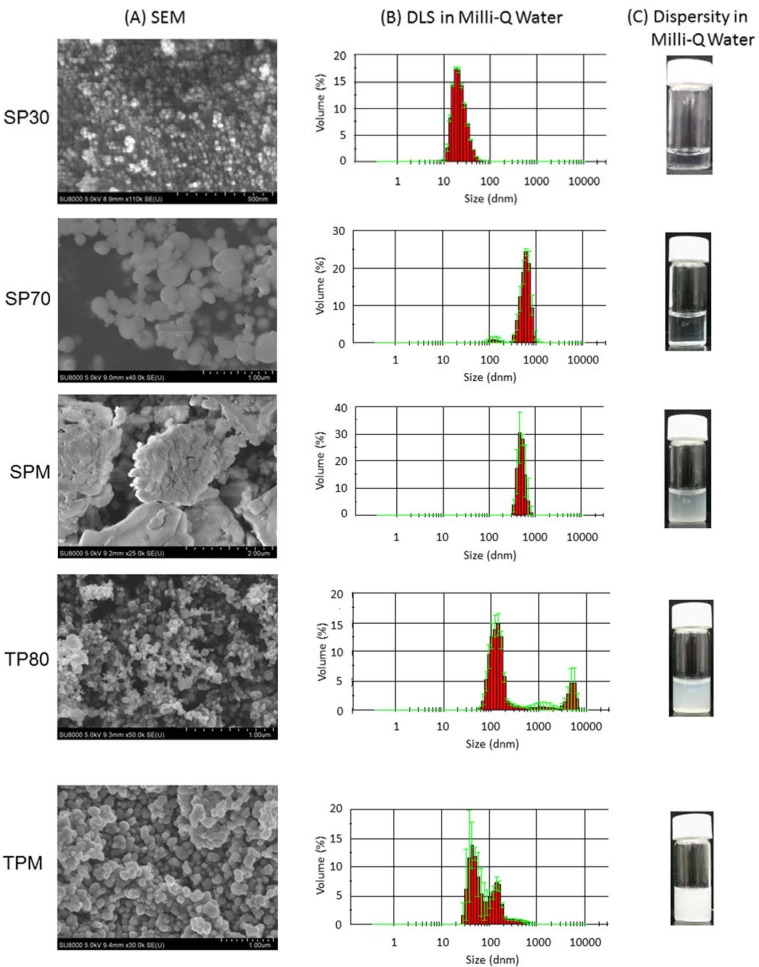
(**A**) SEM images of the particles; (**B**) size distribution histogram of the particles in Milli-Q water (Mean ± S.D., *n* = 3); and (**C**) dispersity in Milli-Q water (1.0 mg/mL).

We studied the properties of SP30, SP70, SPM, TP80, and TPM particles. Scanning electron microscopy (SEM) showed that SP30 particles were uniform size and morphology, whereas the other particles appeared variable size or displayed agglomerated forms (Figure 1A). In addition, measurements of dynamic light scattering (DLS) revealed that the Z-average of dispersed SP30 in water was 28.5 ± 0.03 and its polydispersity index (PDI) (0.116 ± 0.011) was lower than other particles’ PDI (>0.223), suggesting a narrower size distribution (Figure 1B and Table 1). On the other hand, the Z-average of dispersed SPM was 1322.7 ± 112.9 nm and PDI was higher than other particles, suggesting a broader size distribution (Table 1). The other particles (SP70, TP80, and TPM) exhibited 208.5–671.9 in Z-average and 0.223–0.415 in PDI (Table 1). Surface charge data showed that all the particles possessed negative potentials from −26.9 for SP30 to −60.6 for SP70 (Table 1).

SP30 exhibited higher dispersity in Milli-Q water than other particles, and no aggregation was observed (Figure 1C). On the other hand, other particles exhibited less dispersity, in particular, SP70 exhibited agglomeration and sedimentation.

**Table 1:** Physical properties of the dispersed particles in Milli-Q water (supernatant). These data shows mean ± S.D. of two or three measurements.

Particles	Z-Average in Diameter (nm)	PDI	Zeta Potential
**SP30**	28.5 ± 0.03	0.116 ± 0.011	−26.9 ± 0.2
**SP70**	671.9 ± 13.0	0.415 ± 0.028	−60.6 ± 1.3
**SP** **M**	1322.7 ± 112.9	0.698 ± 0.263	−35.3 ± 0.3
**TP80**	208.5 ± 4.3	0.264 ± 0.030	−36.2 ± 0.1
**TP** **M**	210.6 ± 4.7	0.223 ± 0.009	−44.1 ± 1.4

### 2.2. Morphological Effects and Mitochondrial Activity

**Figure 2:**
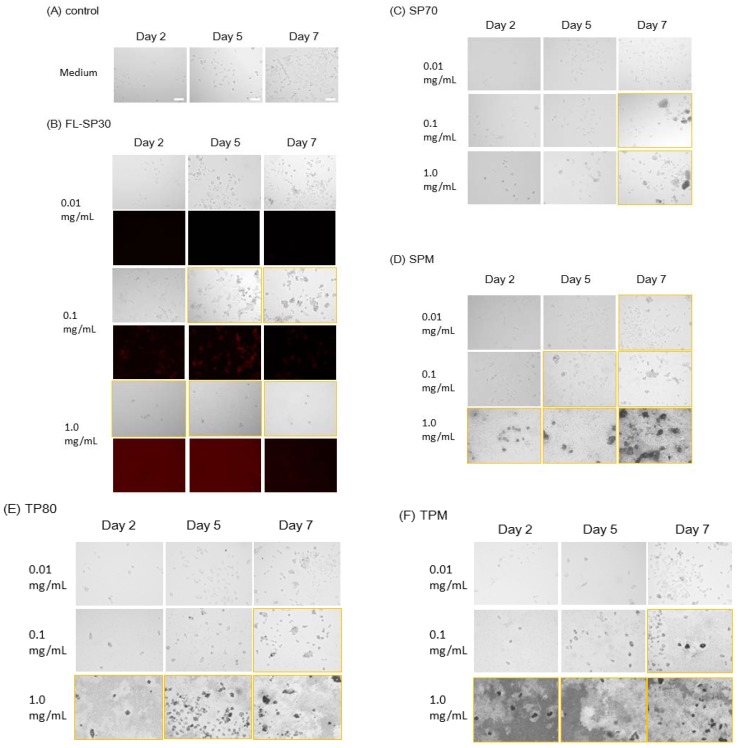
Observation of hNSCs exposed to particles for 7 days. Orange frames indicate cellular aggregation or morphological abnormalities. (**A**) Control; (**B**) FL-SP30; (**C**) SP70; (**D**) SPM; (**E**) TP80; and (**F**) TPM. In the Figure (**B**), bright-field images (**upper**) and fluorescent images (**lower**) exhibit. Since many FL-SP30 attached to the basement of the plate at 1.0 mg/mL, brightness of the fluorescent images (lower) at 1.0 mg/mL was reduced by 50%. Scale bar (white) indicates 100 μm.

We exposed hNSCs to the particles (concentration: 0.01, 0.1, and 1.0 mg/mL) for 7 days. In the control group, which was cultured in normal medium, almost no cellular aggregations and morphological abnormalities were observed (Figure 2A). In contrast, by day 7, all groups exposed to particles at concentrations ≥0.1 mg/mL showed cellular aggregation and morphological abnormalities, such as shrinking or swelling (Figure 2B–F).

Moreover, the fluorescence images showed that fluorescent 30-nm silica particles (FL-SP30) were attached to or incorporated into hNSCs at 0.1 mg/mL (Figure 2B). However, few fluorescent particles were detected at 0.01 mg/mL (Figure 2B). In addition, FL-SP30 particles were also attached to the culture plate at 1.0 mg/mL.

**Figure 3:**
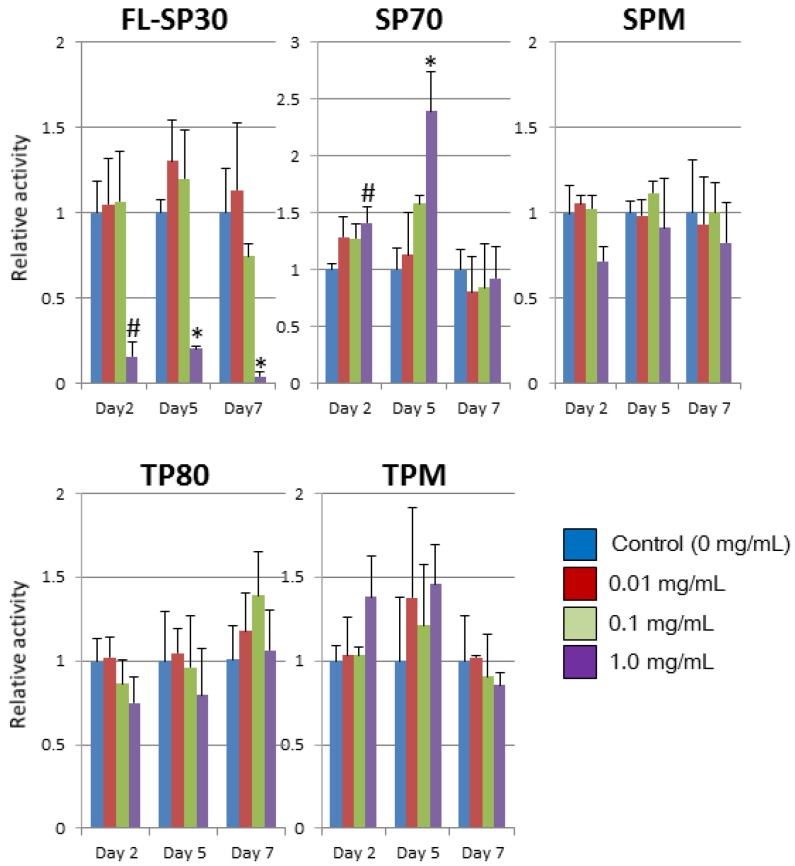
Mitochondrial activity during exposure to particles for 7 days. For avoiding the effects of the interaction between particles and the test reagent/culture plates, the mitochondrial activity was calculated in the subtract absorption, “the absorption of co-cultured well (cell + medium + particles)”—“the absorption of no-cell well (medium + particles)”. The results are presented as mean values (*n* = 3). Error bars represent S.D. The differences between control (0 mg/mL) and particles’ treatments were analyzed with Scheffe’s F test (# *p* < 0.05; * *p* < 0.01).

Further, we studied cytotoxic effects of the particles based on mitochondrial activity during 7 days of exposure to particles (Figure 3). The mitochondrial activity of hNSCs was significantly decreased within 2 days by exposure to 1.0 mg/mL FL-SP30 (*p* < 0.05). The 0.1 mg/mL FL-SP30 tended to reduce the relative activity to 74.5% at day 7. In contrast, other particles did not reduce their relative mitochondrial activities significantly at day 7, although there was variation of mitochondrial activity during the 7 days observed for each particle. For example, the activity of SP70 treatments (1 mg/mL) significantly increased (*p* < 0.05) and the activity of TPM treatments (1 mg/mL) tended to increase (n.s.) at days 2 and 5.

### 2.3. Observation of Cellular Membrane Permeation, Mitochondrial Effects, and Effects on Nuclei

**Figure 4:**
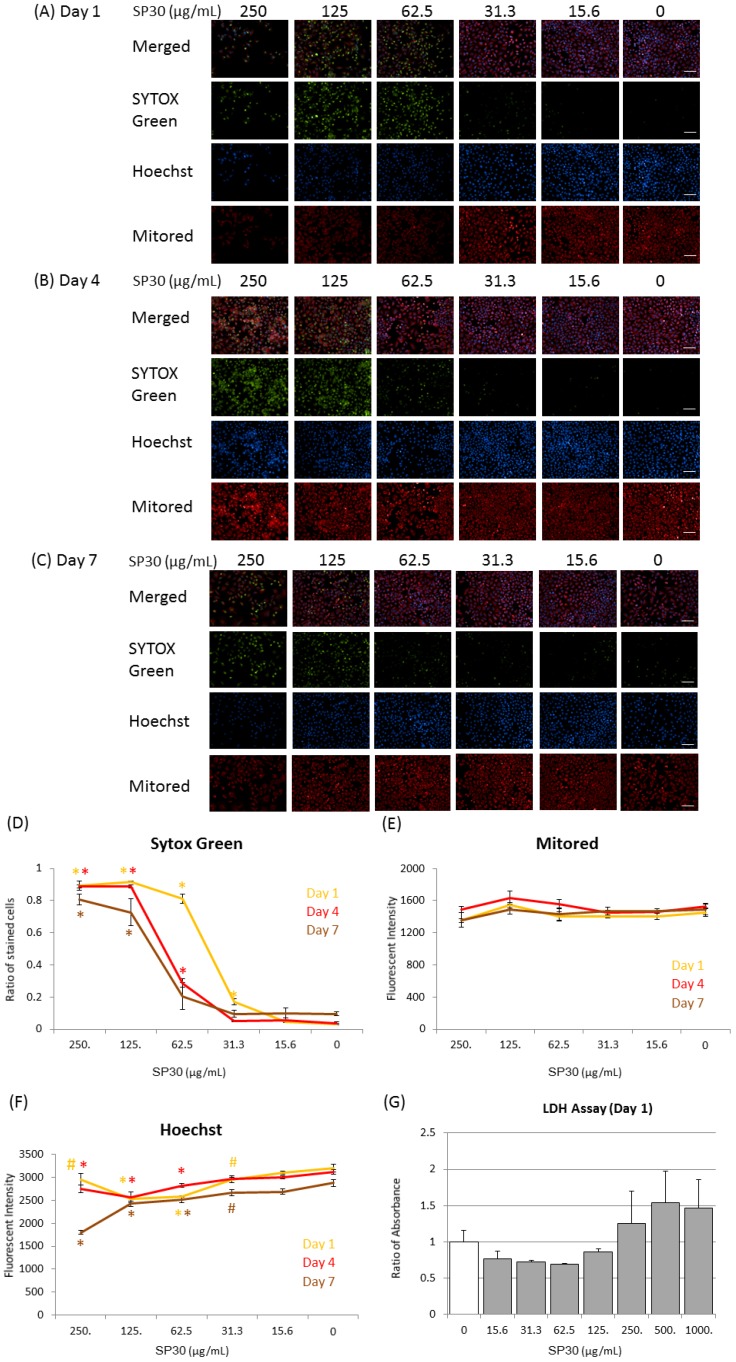
Representative images of nuclei (SYTOX Green and Hoechst) and mitochondria (Mitored) of hNSCs exposed to SP30 (0–250 μg/mL for 7 days), (**A**) Day 1; (**B**) day 4; and (**C**) day 7. Observation was conducted with High Content Imaging System Operetta. From the observation images, around 500–4200 cells were analyzed with the Operetta system in each condition (*n* = 3) (**D**–**F**). (**D**) Ratio of cells stained with SYTOX Green; (**E**) Average intensity of MitoRed staining; (**F**) Average intensity of Hoechst 33342 staining; (**G**) Lactate dehydrogenase (LDH) assay result at day 1 (*n* = 3). Error bars represent S.D. The differences between control (0 μg/mL) and SP30-treatments were analyzed with Scheffe’s F test (# *p* < 0.05; * *p* < 0.01) in each day (**D**–**G**). Scale bar (white) indicates 100 μm.

Because only the mitochondrial activities of hNSCs exposed to FL-SP30 were decreased significantly (*p* < 0.05), we focused on the toxicological effects of 30-nm silica particles. To examine the toxicity in detail, we exposed hNSCs to non-fluorescent 30-nm silica particles (SP30) at several concentrations between 0–250 μg/mL for 7 days and examined the membrane, nuclei, and mitochondrial conditions during this period (Figure 4).

In this experiment, we used SYTOX Green (SG) for nuclear staining to indicate membrane permeability, which is related to viability. At concentrations ≥125 μg/mL, most cells (≥73%) were significantly (*p* < 0.01) stained with SG at 7 days (Figure 4A–D). On day 1, around 81% cells were stained with SG at 62.5 μg/mL (*p* < 0.01) and 17% cells were stained at 31.3 μg/mL (*p* < 0.01) (Figure 4A,D). Interestingly, the recovery, as indicated by less staining, was observed at 62.5 μg/mL on days 4 and 7 (Figure 4D). At 0–31.3 μg/mL, few cells were stained with SG on days 4 and 7. From the plots of SG staining ratio during the 7 days, the IC_50_ on day 1 was between 31.3 and 62.5 μg/mL and the IC_50_ on days 4 and 7 was between 62.5 and 125 μg/mL (Figure 4D).

To confirm the increase in membrane permeability, we conducted a lactate dehydrogenase (LDH) assay. Although no statistically significant difference was found among the SP30 treated groups with Scheffe’s F test, LDH release, which suggested cellular membrane damages, increased at SP30 concentrations ≥250 μg/mL on day 1 (Figure 4G).

On the other hand, the fluorescence intensity of MitoRed, which indicates the electrical potential of mitochondrial activity, showed no significant difference (Figure 4E). 

Furthermore, fluorescent images obtained using the nuclear staining reagent Hoechst 33342, which indicates apoptosis at high staining intensities, showed a concentration-dependent decrease between 31.3 and 125 μg/mL (Figure 4F). At 250 μg/mL, the fluorescence intensity exhibited a tendency to increase at day 1 and 4, whereas it decreased at day 7 (Figure 4F).

### 2.4. Gene Expression of hNSCs Exposed to Particles

**Figure 5:**
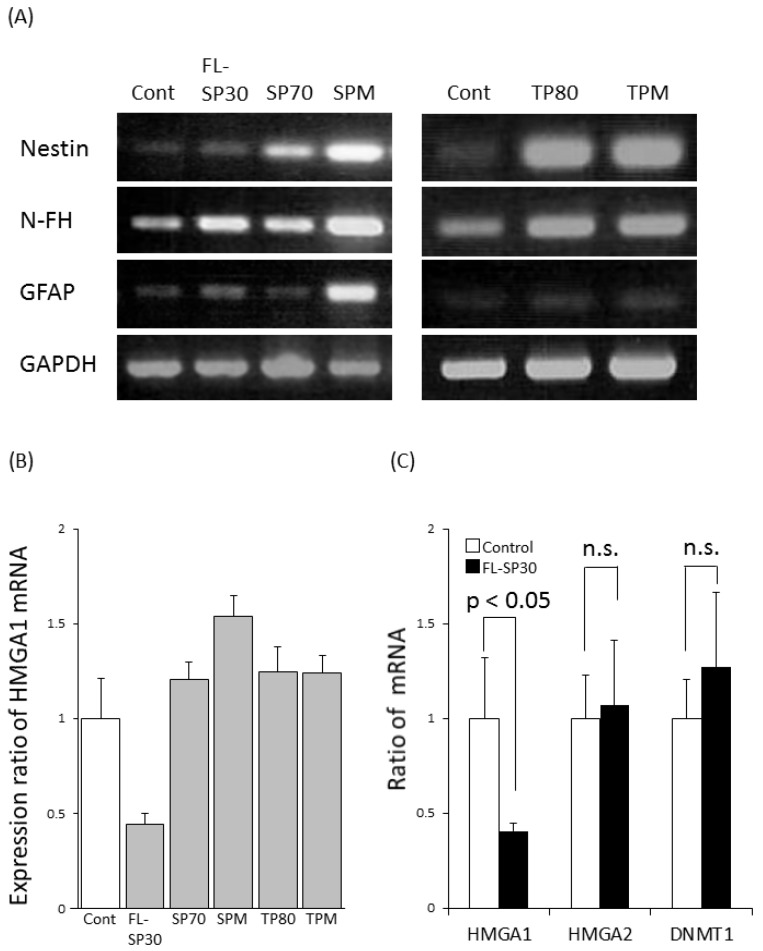
Effects of 0.1 mg/mL particles on gene expression of brain cell markers. (**A**) PCR analyses for differentiation markers (representative data, *n* = 2); (**B**) Real-time PCR analyses for *HMGA1* expression (*n* = 2); (**C**) Effects of FL-SP30 on cellular activity markers in hNSCs (*n* = 3). The differences between Control and FL-SP30 treatments were analyzed by two-sided Student’s *t* test (*p* < 0.05) (**C**). Error bars represent S.D. The n.s. indicates not significantly different from the Control (*p* < 0.05).

Because we found increased membrane permeability of SP30 at the concentrations ≥62.5 μg/mL at day 1 (Figure 4D), we examined the differentiation activity of hNSCs exposed to 0.1 mg/mL. Figure 5A shows the expression profile of genes related to hNSCs differentiation after 24-h exposure of hNSCs to particles at 0.1 mg/mL. We observed the increased expression of *Nestin* (stem cell marker) and neurofilament heavy polypeptide (*N-FH*; neuron marker) in hNSCs after exposure with all the particles (Figure 5A). Moreover, FL-SP30 and SPM exposure increased the expression of glial fibrillary acidic protein (*GFAP*; astrocyte marker). 

Interestingly, the expression of the high mobility group AT-hook 1 (*HMGA1*) gene, which is required for neuronal differentiation during the fetal period [34], was decreased in hNSCs exposed to FL-SP30, which was a different effect than that from other particles (Figure 5B). Focused on FL-SP30 effects, the expression of the *HMGA1* gene was significantly reduced (*p* < 0.05), while the gene expressions of *HMGA2*, which has similar activity to *HMGA1*, and DNA methyltransferase 1 (*DNMT1*), a gene with increased expression in senescent cells, were not significantly affected (Figure 5C).

## 3. Discussion

This study demonstrated that all five particles possessed the ability to affect hNSC morphology at concentrations ≥0.1 mg/mL (Figure 2). In addition, we found that 30-nm silica particles significantly affected the mitochondrial activity of hNSCs at 1.0 mg/mL (Figure 3) and membrane permeability at concentrations ≥62.5 μg/mL (Figure 4).

These toxicological concentrations may not be realistic in the brain at single exposure because another *in vivo* study suggested that the ratio of quantum dots accumulation in the brain was approximately 0.1% of the dose [23]. However, continuous or long-term exposure may increase the concentration in the brain. Therefore, these thresholds may indicate the potential toxicity of accumulated particles for long-term usage or continuous exposure. Moreover, these concentrations will provide a reference for the maximum particles dose for NSCs and will be useful in the fields of occupational and consumer health until the long-term influences *in vivo* at lower concentration are revealed.

Toxicological effects were detected at 0.1 and 1.0 mg/mL, regardless of the main particle sizes or materials (Figure 2). Moreover, the concentration that induced cellular aggregation appeared to be different by the types of particles. As for the relevance of particles sizes to up-taken particles, Sakai *et al.* showed that the fluorescent polystyrene nanoparticles (22 and 100 nm) were taken up in rat pheochromocytoma (PC12) cells more than microparticles (1000 nm) both in particle number and weight [37]. Additionally, they showed that all the particles decreased cellular viability (mitochondrial activity) at 10% concentration of particles for 24-h culture and then 22-nm particles (0% of viability) was more cytotoxic than other 100 nm (73%) and 1000 nm (54%) particles [37]. Our data also showed that all the nano and microparticles affected cellular morphology ≥0.1 mg/mL within 7 days (Figure 2) and 30-nm silica particles decreased the mitochondrial activity more than other particles (Figure 3). With regard to the toxicity of nano-silica particles (21, 48, and 86 nm), Ye *et al.* reported that the cytotoxicity of particles depended on the size, concentration, and time in the L-02 human hepatic cell line [38]. These results led to the hypothesis that (1) the cytotoxicity mechanism of particles may be different in the particles’ sizes, especially nanoparticles or microparticles; and (2) around 21–30 nm particles may be more cytotoxic than other larger nanoparticles.

How did the 30-nm silica particles affect hNSCs? One important factor was membrane damage by 30-nm silica particles (Figure 4). Recent toxicological studies on silica particles have suggested that the ROS production due to silica particles contributes to the toxicological effects. For example, Kim *et al.* reported that several silica particles smaller than 20 nm increased ROS production dose-dependently in the SH-SY5Y human neural cell line [39]. Park *et al.* indicated that 20-nm silica particles caused greater cell damage and ROS production than 100-nm particles in the HaCaT human keratinocyte cell line [40]. Because ROS-related lipid peroxidation has also been reported using nano-silica particles [38], ROS generated by 30-nm silica particles used in this study may induce membrane damages in NSCs. Actually, although we tried to detect ROS with 2',7'-dichlorofluorescin diacetate, we have not yet obtained the evidence of ROS generation in hNSCs by 30-nm silica particles (data not shown). In future studies, the mechanism should be investigated.

We also investigated osmotic pressure as another mechanism underlying membrane damage. However, osmotic pressure may not be relevant to membrane permeability in the present study, because the pressure of the solution was not affected significantly (around detection limit: ±0.001 mOsm/kg) at the SP30 concentration of 1.0 mg/mL or less (data not shown).

Observations of nuclei and mitochondria during exposure to SP30 showed a significant increased rate of SG staining, indicating disrupted cell membranes and necrosis (Figure 4). These results also revealed that the threshold concentration for acute membrane permeation during 24 h was 62.5 μg/mL. In addition, from the observations of FL-SP30, we detected attachment or incorporated particles around hNSCs at 0.1 mg/mL but not at 0.01 mg/mL (Figure 2B). These data also supported an acute increase in membrane permeability above the threshold concentration and did not conflict the report by Song *et al.*, which showed that proliferations and viabilities of hNSCs were not affected by the co-culture of several nanoparticles at 25 μg/mL for 24 h [35]. Interestingly, a repair mechanism or resistance to membrane permeation was also suggested, because the SG staining ratio at 62.5 μg/mL decreased at days 4 and 7 (Figure 4D).

Our findings on the gene expression of differentiation markers suggest that exposure to all the particles in this study increased *Nestin* and *N-FH* expression (Figure 5A). Moreover, we found an increase in the expression of *GFAP* after exposure to FL-SP30 and SPM at 0.1 mg/mL (Figure 5A). These results suggested that exposure to particles (irrespective of their size) may lead not only to self-renewal but also to promotion of neural differentiation spontaneously, maybe due to the toxicological effects. Furthermore, FL-SP30 and SPM at 0.1 mg/mL have the potential to lead to astrocyte differentiation of NSCs. Similar to our results, another study showed that the mouse NSC cell line C17.2, exposed to 0.15 mg/mL titanium oxide nanoparticles coated with SiO_2_ (80–100 nm in diameter) for 7 days, was induced to differentiate into neurons [41]. Therefore, exposure to silica or titanium particles may affect the differentiation of NSCs.

Finally, we found that exposure to FL-SP30 decreased the gene expression of *HMGA1* in hNSCs slightly (Figure 5C), which is required for neural development [34]. This result suggested that FL-SP30 at 0.1 mg/mL may decrease the neurogenesis of hNSCs. On the other hand, we have found the increase of *NF-H* gene expression, neuronal marker (Figure 5A). Therefore, in a future study, the activity of neurons or the ratio of neurons differentiated from hNSCs exposed to the 30-nm or smaller than 100-nm silica particles should be examined. Notably, our previous study showed the same FL-SP30 (used in this report) indicated a slight penetration ability into brain area in the blood brain barrier model *in vitro* [30]. Moreover, other researchers showed silica particles smaller than 100 nm crossed the blood brain barrier [42] and placenta [29] *in vivo*.

## 4. Experimental Section

### 4.1. Particles

Silicon dioxide, 70 nm (Wako Pure Chemical Industries, Osaka, Japan), silicon dioxide, −325 mesh (Sigma-Aldrich, St. Louis, MO, USA), sicastar, non-coating, 30 nm (Micromod Partikeltechnologie GmbH, Rostock, Germany), and sicastar-redF, non-coating, 30 (Micromod Partikeltechnologie GmbH) were used as silica particles, namely SP70, SPM, SP30 and FL-SP30, respectively. Titanium (IV) oxide, 80 nm (Wako Pure Chemical Industries, Osaka, Japan), Titanium (IV) oxide, anatase, powder, −325 mesh (Aldrich, St. Louis, MO, USA) were used as titanium oxide particles, namely TP80 and TPM, respectively. 

Since the 30 nm silica nanoparticles was dispersed in an aqueous solution, we centrifuged the solution at 65,000 rpm for 8 h at room temperature (Himac CP80MX, Hitachi, Tokyo, Japan). The obtained precipitate was further dried by a centrifugal evaporator (CVE200D, Tokyo Rika Kikai, Tokyo, Japan) for SEM measurement. For investigation of physical properties about dispersed particles in Milli-Q water (Merck Millipore, Billerica, MA, USA) (Table 1), we measured size in DLS, PDI, and zeta-potential, two (zeta potential of SP30) or three times (other measurement) with Zetasizer Nano ZS (Malvern Instruments, Worcestershire, UK) in default measurement mode. We showed average data in the Table 1. After experiments, we measured the pH of Milli-Q with pH indictor paper (Whatmann pH 1–11 indicator paper, GE Healthcare UK Ltd., Buckinghamshire, UK), which indicated pH 5. Additionally, we observed the particles with ultra-high resolution scanning electron microscope SU8000 (Hitachi, Tokyo, Japan). 

### 4.2. Cell Culture and Morphology Observation of Human Neural Stem Cell Line

HB1.F3 human neural stem cell line described in references [43,44,45] was cultured in high-glucose DMEM (Life Technologies, Carlsbad, CA, USA) with 10% fetal bovine serum and penicillin streptomycin, in culture plates under the condition humidified 5% CO_2_ at 37 °C. Culture plates, Costar 24 Well Clear TC-Treated Multiple Well Plates, Individually Wrapped, Sterile (Corning Incorporated, Corning, NY, USA), Corning 96 Well Clear Flat Bottom TC-Treated Microplate, Individually Wrapped, with Low Evaporation Lid, Sterile (Corning Incorporated, Corning, NY, USA), or ViewPlate-96 Black, Optically Clear Bottom, Tissue Culture Treated, Sterile, 96-Well with Lid (PerkinElmer, Waltham, MA, USA) were used for the hNSC cultures and particles treatments. All the particles were added into the culture medium at the indicated concentrations, quickly after dispersed in Milli-Q water.

For morphology observation, 4 × 10^4^ hNSCs were added into each well of the 24-well plate (Corning Incorporated, Corning, NY, USA). Forty-eight hours after seeding the cells, each well was treated with the indicated doses of particles for 48–168 h (Day 2: 48 h; Day 5: 120 h; Day 7: 168 h in Figure 2), with particles in concentration of 0, 0.01, 0.1, or 1.0 mg/mL. Each experiment was conducted two times independently. The co-cultured hNSCs in Figure 2 were observed with BZ-9000 (Keyence, Osaka, Japan) after trypan blue staining. In the fluorescent images in Figure 2B, brightness of the fluorescent images at 1.0 mg/mL was reduced by 50% with PowerPoint 2013 (Microsoft, Redmond, WA, USA) for visibility, since 30-nm fluorescent silica particles attached to the basement of the plate. Background brightness of other non-fluorescent wells was adjusted using PowerPoint for comparing colors. 

### 4.3. Mitochondrial Activity Assay

The effect of particles on the hNSC mitochondrial activity was measured using the Cell Counting Kit-8 (CCK-8, Dojindo Molecular Technologies, Kumamoto, Japan) as previously described [16,37,46]. Briefly, 1 × 10^4^ hNSC were added into each well of the 96-well plate above (Corning Incorporated, Corning, NY, USA). Forty-eight hours after seeding the cells, each well was treated with indicated doses of particles for 48–168 h (Day 2: 48 h; Day 5: 120 h; Day 7: 168 h in Figure 3), with particles in concentration of 0, 0.01, 0.1, or 1.0 mg/mL. Each experiment was conducted three times independently. Then the CCK-8 solution was added to each well. After cells were incubated for another 30–60 min, the absorbance at 450 nm was measured using a microplate reader (680XR, Bio-Rad, Hercules, CA, USA). The mitochondrial activity was calculated in the subtracted absorption, “the absorption of co-cultured well (cell + medium + particles)”—“the absorption of no-cell well (medium + particles)”, because some particles, themselves, may have the potential to increase the absorption due to their reducing potential and attachment of plates. Each experiment was done in triplicates. The differences between control (0 mg/mL) and particles’ treatments were analyzed with Scheffe’s F test (# *p* < 0.05; * *p* < 0.01) (Figure 3).

### 4.4. Observation of Cytotoxic Effects with Nuclear and Mitochondria Staining

hNSC (1 × 10^4^ cells) were added to each well of the 96-well plate mentioned above (PerkinElmer, Waltham, MA, USA). From 48 h after seeding the cells, each well was treated with 0–250 μg/mL of SP30 for 24–168 h (Day 1: 24 h; Day 4: 96 h; Day 7: 168 h) in Figure 4. SYTOX Green 11 Nucleic Acid Stain (Life Technologies, Carlsbad, CA, USA) as lower membrane permeability reagent, and Hoechst 33342 solution (Dojindo Molecular Technologies, Kumamoto, Japan) as higher membrane permeability reagent were used for nucleic acid staining. Mitochondria were stained with MitoRed (Dojindo Molecular Technologies, Kumamoto, Japan). After fixation, these stained hNSC were observed with High Content Imaging System Operetta (PerkinElmer, Waltham, MA, USA). Each particles treatments were conducted three times independently and whole samples were observed at one day with the Operetta. The differences between control (0 μg/mL) and SP30-treatments (0–250 μg/mL) were analyzed with Scheffe’s F test (# *p* < 0.05; * *p* < 0.01) in each day (Figure 4D–F).

### 4.5. Lactate Dehydrogenase (LDH) Assay

For the measurement of LDH release from hNSC cells exposed to the indicated SP30 for 24 h (three independent experiments), we used an LDH Cytotoxicity Detection Kit (Takara Bio, Shiga, Japan) as an indicator for plasma membrane leakage as described in our previous paper [16]. The 490 nm absorption of formazans as an indicator of LDH releases were measured with a plate reader. The differences between control (0 μg/mL) and SP30-treatments (0–1000 μg/mL) were analyzed with Scheffe’s F test (# *p* < 0.05; * *p* < 0.01) (Figure 4F).

### 4.6. PCR and Real-Time PCR Analyses

We performed PCR analyses and real-time PCR (RT-PCR) analyses of hNSCs to identify the expressed gene after 24 h-exposure of a 0.1 mg/mL of particles solution. Total RNAs were prepared from the hNSCs which were treated with particles in 0.1 μg/mL (24 well plate) for 24 h, using ISOGEN II (Nippongene, Tokyo, Japan). Each experiment was conducted two or three times independently. These RNAs were reverse-transcribed with ReverTra Ace qPCR RT Master Mix with gDNA Remover (Toyobo, Osaka, Japan). 

PCR assays were conducted with Quick Taq HS DyeMix (Toyobo, Osaka, Japan) reagent and the amplified samples were loaded to 1.5% agarose gels (Agarose S, Nippongene, Tokyo, Japan). RT-PCR analyses were conducted with FastStart Universal SYBR Green Master (ROX) reagent (Roche, Basel, Switzerland) using the 7300 Real Time PCR System (Applied Biosystems, Carlsbad, CA, USA). 

For PCR and RT-PCR primers, Perfect Real Time Primer (Takara Bio, Shiga, Japan) were used for amplifications as below, HA042877 for *Nestin*, HA151016 for neurofilament, heavy polypeptide (*N-FH*), HA157438 for glial fibrillary acidic protein (*GFAP*), HA180245 for high mobility group AT-hook 1 (*HMGA1*), HA08633 for high mobility group AT-hook 2 (*HMGA2*), and HA126052 for DNA (cytosine-5-)-methyltransferase 1 (*DNMT1*). For glyceraldehyde-3-phosphate dehydrogenase (*GAPDH*) amplification, Perfect Real Time Primer HA067812 and custom primer sets synthesized in Greinar Japan, Tokyo, Japan (forward 5'-CATGACCACAGTCCATGCCATCACT-3', reverse 5'-TGAGGTCCACCACCCTGTTGCTGTA-3' [45]) were used in RT-PCR analyses and PCR analyses, respectively. For the data analysis, the differences between control and FL-SP30 treatments were analyzed by two-sided Student’s *t* test (Figure 5C).

### 4.7. Statistical Analyses

Statistical analyses mentioned above were performed with Microsoft Office Excel 2007 (Microsoft, Redmond, WA, USA) and the add-in software Statcel 3 (OMS publishing Inc., Saitama, Japan).

## 5. Conclusions

This study demonstrated that all the silica and titanium oxide particles both smaller and greater than 100 nm (1) had the ability to affect hNSCs morphology at the concentration of ≥0.1 mg/mL during 7 days of culture and (2) affected the gene expression of differentiation markers at a concentration of 0.1 mg/mL. Although these toxicological concentrations were higher than the expected concentrations in the brain resulting from the injection/exposure *in vivo* experiments in a short period, these thresholds may indicate the potential toxicity of accumulated particles for long-term usage or continuous exposure.
